# Are ribosomal DNA clusters rearrangement hotspots? A case study in the genus *Mus *(Rodentia, Muridae)

**DOI:** 10.1186/1471-2148-11-124

**Published:** 2011-05-13

**Authors:** Benoîte Cazaux, Josette Catalan, Frédéric Veyrunes, Emmanuel JP Douzery, Janice Britton-Davidian

**Affiliations:** 1 Institut des Sciences de l'Evolution, UMR5554 CNRS/Université Montpellier II, Montpellier, France

## Abstract

**Background:**

Recent advances in comparative genomics have considerably improved our knowledge of the evolution of mammalian karyotype architecture. One of the breakthroughs was the preferential localization of evolutionary breakpoints in regions enriched in repetitive sequences (segmental duplications, telomeres and centromeres). In this context, we investigated the contribution of ribosomal genes to genome reshuffling since they are generally located in pericentromeric or subtelomeric regions, and form repeat clusters on different chromosomes. The target model was the genus *Mus *which exhibits a high rate of karyotypic change, a large fraction of which involves centromeres.

**Results:**

The chromosomal distribution of rDNA clusters was determined by *in situ *hybridization of mouse probes in 19 species. Using a molecular-based reference tree, the phylogenetic distribution of clusters within the genus was reconstructed, and the temporal association between rDNA clusters, breakpoints and centromeres was tested by maximum likelihood analyses. Our results highlighted the following features of rDNA cluster dynamics in the genus *Mus*: i) rDNA clusters showed extensive diversity in number between species and an almost exclusive pericentromeric location, ii) a strong association between rDNA sites and centromeres was retrieved which may be related to their shared constraint of concerted evolution, iii) 24% of the observed breakpoints mapped near an rDNA cluster, and iv) a substantial rate of rDNA cluster change (insertion, deletion) also occurred in the absence of chromosomal rearrangements.

**Conclusions:**

This study on the dynamics of rDNA clusters within the genus *Mus *has revealed a strong evolutionary relationship between rDNA clusters and centromeres. Both of these genomic structures coincide with breakpoints in the genus *Mus*, suggesting that the accumulation of a large number of repeats in the centromeric region may contribute to the high level of chromosome repatterning observed in this group. However, the elevated rate of rDNA change observed in the chromosomally invariant clade indicates that the presence of these sequences is insufficient to lead to genome instability. In agreement with recent studies, these results suggest that additional factors such as modifications of the epigenetic state of DNA may be required to trigger evolutionary plasticity.

## Background

The extensive advances in comparative cytogenomics in recent years have considerably enhanced our knowledge of the evolution of mammalian genomic architecture [1]. In particular, the identification of syntenic associations of homologous chromosomal segments has led to the delimitation of breakpoint sites, i.e., regions where genome synteny has been disrupted by chromosomal rearrangements. Complementary to the cytogenetic approach, whole genome comparisons between different mammalian groups have highlighted the extensive reuse of breakpoints during chromosome evolution supporting the notion of evolutionary breakpoints as hotspots of genome repatterning [2]. This predisposition of certain genomic regions to instability has been formalized as the 'fragile breakage model' [3]. Moreover, comparative genomics, particularly in primates, has provided greater resolution of breakpoints. These studies have shown that breakpoint regions are enriched with different types of repetitive sequences such as SINEs, LINEs, LTRs and, in particular, segmental duplications [2,4-6]. The presence of repetitive sequences at evolutionary breakpoints is thought to be related to the role tandem repeats play as a substrate for non-homologous recombination (i.e., exchanges between two different chromosomes) thereby promoting chromosomal rearrangements [7]. However, several analyses suggest that rearrangement breakpoints and repeat sequences (flanking regions of segmental duplications) share similar physicochemical properties (high DNA flexibility and low stability) characteristic of fragile sites [1,8]. Centromeric and subtelomeric domains appear as the key regions in chromosome evolution, since both accumulate repeat sequences, harbour many breakpoints and engage in non-homologous recombination [9,10]. Several studies have shown a significant association between breakpoint reuse and centromere repositioning in different mammalian groups such as Marsupialia, Muridae, Equidae, and Primates [2,11-13], underscoring the pivotal role of these genomic regions in chromosome restructuring.

The rRNA genes represent another family of tandem repeat sequences. These genes code for the ribosomal subunits that are essential for the cellular translation machinery. In mammals, each unit is composed of three genes coding for 18S, 5.8S and 28S ribosomal RNA; these genes are separated by two intergenic spacers and an external transcribed spacer [14]. Whereas the sequences of spacers are generally highly divergent, the ribosomal coding elements show in some regions a remarkable sequence conservation within species and even among distantly related organisms [15]. The tandem repeats of units are further organized into clusters present on one to several chromosome pairs where they are most often located in pericentromeric or subtelomeric regions [4,16-18]. Several studies have highlighted the species-specific chromosomal distribution of rDNA clusters even between closely related species, suggesting that the location of rDNA clusters can rapidly change through transposition. The rDNA clusters therefore show several features in common with breakpoint regions: they are tandemly repeated; they are generally located in pericentromeric and subtelomeric regions; they transpose; they are subject to high rates of intra- and inter-chromosomal recombination. Indeed, the coincidence of rDNA clusters with chromosomal breakpoints has been highlighted *in vitro *in plants [19]. Here, we investigate the evolutionary dynamics of rDNA clusters in relation to genome reshuffling in an emblematic mammalian model, the genus *Mus *which includes the house mouse.

*Mus *is an ideal biological model to investigate processes of chromosomal evolution since it exhibits one of the highest rates of karyotypic repatterning documented in mammals [20]. The genus is species-rich with more than 40 species distributed among four subgenera [21]. These include the subgenus *Coelomys *(shrew mice) with four species and a South-East Asian distribution, the subgenus *Pyromys *(spiny mice) with five species restricted to the Indian subcontinent, and the African subgenus *Nannomys *(pygmy mice) the most species-rich (with 18 species) and karyotypically diverse of the four subgenera [21,22]. Finally, the Eurasian subgenus *Mus *comprises 16 species, one of which is the house mouse [21,23]. Comparative cytogenomics has revealed a 10- to 30-fold acceleration in chromosomal change that was coincidental with the subgeneric cladogenesis, followed by a remarkable stasis in the subgenus *Mus *in which all 16 species share the same 2n = 40 karyotype. Moreover, studies have shown that more than half of the observed rearrangements involved centromeres (reactivation of latent sites or neocentromerization events) [13,24].

The aim of the present study was to investigate if the evolutionary dynamics of rDNA clusters played a role in the extensive genomic reshuffling of the genus *Mus*. To do so, we first established the variation in location and number of the rDNA clusters between species belonging to different subgenera. Second, we used the available molecular phylogenies to reconstruct and track the evolution of these clusters in the genus. Finally, building upon the existing chromosomal phylogeny, we tested the temporal association between rDNA clusters, breakpoint regions, and centromeres.

## Methods

### Material

The 19 species and subspecies as well as the number of specimens studied are listed in Table 1. Animals were either obtained from the Conversatoire Génétique de la Souris Sauvage (Institut des Sciences de l'Evolution, Montpellier, France) or collected in the wild. Four additional species (*Mus booduga, M. terricolor, Apodemus sylvaticus, Rattus rattus*) for which rDNA cluster data were available, were included in the analyses [25-27].

**Table 1:** Chromosomal distribution of 18S and 28S rDNA clusters in the genus *Mus*

Species	N	Locality	2n	**Chromosomes **^**a**^	**N° of sites **^**b**^	Reference
**Outgroup**						

*Rattus rattus*			38	5, 8, 16	6	[25]
*Apodemus sylvaticus*			48	7*, 8*, 11*, 12*, 15*, 16*, 21*, 22*	16	[27]

***Subgenus Coelomys***						
*M. pahari*	1	PAH ^d^	48	1, 2, 3, 4, 5, 7, 8, 10, 11, 12, 13, 14, 15, 16, 17, 18, 19, 20, 21, 22, 23	42	this study

***Subgenus Pyromys***						
*M. plathytrix*	1	PTX ^d^	26	5, 8, 12	6	this study, [26]

***Subgenus Nannomys***						
*M. matthey*	2	laboratory strain	36	1, 4, 8, 11, 13, 14, 17	14	this study
*M. musculoides *^*c*^	1	Cameroun	18	4.13, 8.15	4	this study
*M. minutoides *^*c*^	1	South Africa	18	4.7, 12.17, 13.16, 14.15	8	this study
*M. indutus*	1	South Africa	36	14, 15, 17	6	this study
*M. haussa*	1	Mali	36	15	2	this study

***Subgenus Mus***						
*M. caroli*	2	Thailand	40	1, 2, 3, 4, 5, 6, 7, 8, 9, 10, 11, 12, 13, 14, 15, 16, 17, 18, 19, X	40	this study
*M. cervicolor*	2	Thailand	40	4, 5, 6, 7, 8, 9, 10, 11, 12, 13, 14, 15, 16, 17, 18, 19	32	this study
*M. cooki*	1	COK ^d^	40	7, 8, 9, 10, 11, 12, 13, 14, 15, 16, 17, 18, 19	26	this study
*M. fragilicauda*	3	Thailand	40	1, 2, 3, 4, 5, 6, 7, 8, 9, 10, 11, 12, 13, 14, 15, 16, 17, 18, 19	38	this study
*M. famulus*	2	India	40	2, 3, 4, 5, 6, 7, 8, 9, 10, 11, 12, 13, 14, 15, 16, 17, 18, 19	36	this study
*M. spicilegus*	2	XBJ ^d^	40	5, 6, 8, 16, 19	10	this study,[26]
*M. spretus*	3	Fr, Sp, Morocco	40	4*, 13*, 19*	6	this study, [41]
*M. macedonicus*	2	Israël	40	3, 4, 5, 8, 12, 16, 19	14	this study, [26]
*M. cypriacus*	3	Cyprus	40	1, 4, 11, 15, 16, 17, 18, 19	16	this study
*M. m. castaneus*	1	Thailand	40	4, 8, 9, 10, 11, 12, 15, 16, 18, 19	20	this study, [42]
*M. m. musculus*	1	Poland, Denmark	40	4, 8, 10, 11, 12, 15, 16, 17, 19	18	this study, [42]
*M. m. domesticus*	2	France	40	12, 15, 16, 18, 19, 4*	12	this study,[42]
*M. booduga*			40	1, 3, 4, 6, 8, 9, 11, 12, 13, 14, 15, 16, 17, 18	28	[26]
*M. terricolor*			40	4, 6, 7, 12, 15, 17, 18, 19	16	[26]

*: rDNA clusters are located at the distal end of chromosomes

a: chromosome numbers refer to the karyotype of the species.

b: number of sites refers to the number of chromosome pairs bearing rDNA clusters

c: chromosomes separated by a period are those involved in a Robertsonian fusion

d: strain of the Conservatoire Génétique de la Souris Sauvage

N: number of specimens studied

Fr: France

Sp: Spain

### Chromosomal analyses

For all taxa, mitotic metaphases were obtained by the air-drying method from bone marrow cells after yeast stimulation [28]. Identification of chromosomes was performed by DAPI-banding following the nomenclature of Cowell [29] for the subgenus *Mus*, Veyrunes *et al. *[22,24] for the subgenera *Nannomys *and *Coelomys*, and Matsubara *et al. *[26] for the subgenus *Pyromys*. At least five metaphases per specimen were analysed. All observations were made with a Zeiss Axiophot fluorescence microscope equipped with an image analyser (Cytovision 3.93.2, Genetix).

### Fluorescence in-situ hybridization

The chromosomal location and number of rDNA clusters was determined by fluorescence *in-situ *hybridization (FISH). Cloned gene fragments of the house mouse 28S rDNA [BE-2-pSP64, 1.5kb; [30]] and 18S rDNA [SalC-pSP64, 2kb; [31]] were labelled separately with Digoxigenin-11-dUTP by nick translation according to the Roche Protocol and added to the same hybridization solution. The chromosome slides were treated with RNase for 1 hour at 37°C to remove cellular rRNA, dehydrated in a series of ethanol washes and then air-dried. They were denatured for 2 min at 72°C in 70% formamide, 2XSSC, dehydrated in a series of ice-cold ethanol washes and air-dried. The probes were denatured for 10 min at 72°C. The chromosome slides were hybridized overnight with the two probes (150 ng/slide). The slides were washed at 37°C for 2 min in 2XSSC and 4XT (4x SSC, 0.05% Tween 20, pH = 7) before they were incubated with FITC conjugated with anti-digoxigenin antibody (Roche). The slides were mounted in a Vectashield antifade solution containing DAPI (4', 6'-diamidino-2-phenylindole; Vector Laboratories). A cluster was considered as present on a chromosome pair when a signal was observed on at least one of the homologs. Three positions were possible: i) pericentromeric when the cluster was observed on the proximal region of the chromosome, i.e., adjacent to the centromere, ii) subtelomeric when on the distal end of the chromosome, and iii) interstitial when neither proximal nor distal.

### Phylogenetic inference of rDNA cluster evolution

**Figure 1:**
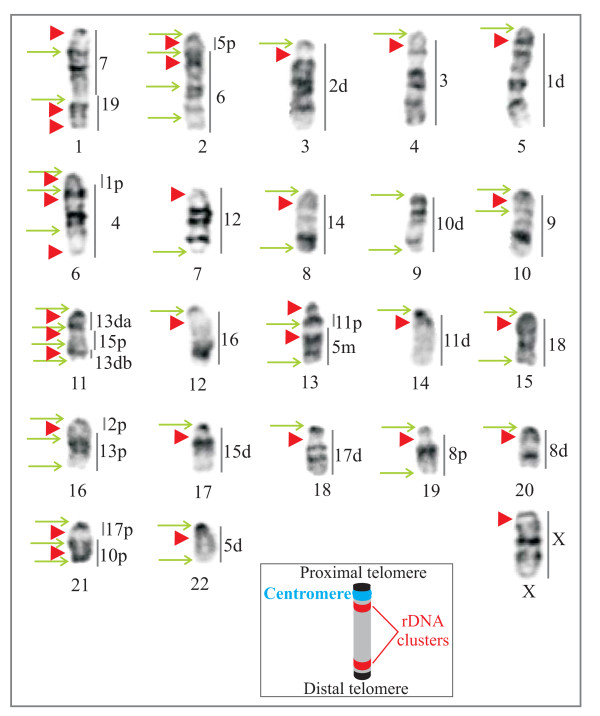
**Ancestral karyotype of the genus *Mus *with location of breakpoints and rDNA clusters **[24]. All chromosomes are acrocentric. The segments orthologous to the house mouse chromosomes are indicated at the right of each chromosome; p, m, d refer to the proximal, median and distal segments of the chromosome, and a and b to unidentified subchromosomal segments. The green arrows point to breakpoints and the red arrowheads to rDNA clusters. The insert shows a schematic acrocentric chromosome with the two possible locations of rDNA clusters (red): (i) pericentromeric i.e. adjacent to the centromere (blue) and (ii) subtelomeric i.e. close to the distal telomere (black).

The determination of the phylogenetic distribution of the rDNA clusters throughout the genus *Mus *first required that the chromosomal orthology among all taxa be established. For this, we relied on the comparative chromosome maps for the subgenera established by Veyrunes *et al. *[24] based on Zoo-FISH and the chromosome nomenclature of the house mouse as reference. The Veyrunes *et al. *[24] study concluded that the ancestral *Mus *karyotype comprised 30 syntenic segments (Figure 1). A matrix was then constructed using these 30 segments as characters and the following states: the presence or absence of rDNA clusters on the proximal region of the segment was coded 1 or 0 respectively, and a distal localisation as 2. *Apodemus sylvaticus *and *Rattus rattus *were used as outgroups in all analyses. The chromosomal homology between these species and the house mouse was previously determined by Matsubara *et al. *[27] and Cavagna *et al. *[25] respectively. However, as Matsubara *et al. *[27] did not discriminate a distal and proximal segment corresponding to mouse chromosome 1 in *A. sylvaticus*, the presence or absence of clusters on these segments was coded as "?".

Next, a reference tree was needed providing a topology and branch lengths and including all of the species studied. As none of the published phylogenies met this requirement, we reconstructed a molecular phylogeny of the genus *Mus *using the nuclear interstitial retinol-binding protein 3 (RBP3 = IRBP) and mitochondrial cytochrome b gene sequences. These data were available in GenBank for all species (Table 2) except *Mus cypriacus: *the full-length sequence (1140 bp) of this gene was sequenced for *M. cypriacus *following Montgelard *et al. *[32] [EMBL: FR751074]. The sequences were aligned using Bioedit (v.7.0.5). The best fitting model was determined by Modeltest [33] to be the GTR+G+I model (-LnL = 11707.61; A = 26%, C = 30%, G = 21%, T = 23%, proportion of invariable sites = 62%, gamma shape parameter = 0.95). The phylogenetic analyses were performed on a constrained topology and the combined dataset (the missing data were coded by "?") using maximum likelihood (ML) as implemented in PAUP* v.4b.0. Two nodes of the topology were constrained according to published data: (i) the relationships between the subgenera were unambiguously resolved by Veyrunes *et al. *[24] with the subgenus *Coelomys *diverging first, followed by *Nannomys *as the sister group of the *Mus*-*Pyromys *clade.; (ii) *M. cypriacus *and *M. macedonicus *were considered as sister-species as proposed by Cucchi *et al. *[34]. According to the highest-likelihood topology, the subgenus *Mus *was divided into three clades: an Southeast Asian group with three species (*M. caroli, M. cervicolor *and *M. cooki*), an Indian group (*M. terricolor, M. booduga, M. fragilicauda *and *M. famulus*), and a Palearctic group (*M. spretus, M. spicilegus, M. macedonicus, M. cypriacus*, and the three subspecies of *M. musculus *[35,36]). The sister-species relationships within some of the Paleartic taxa were not resolved. However, slight changes in the topology of the reference tree did not affect the results, since the chromosomal distribution of clusters within these groups was quite homogeneous.

**Table 2:** Accession number of genes for each species

Species	Cytochrome B	IRBP
*R.rattus*	160688818, [80]	__
*A. sylvaticus*	AB033695, [81]	AB032863, [82]
*M. pahari*	AY057814, [83]	AJ698893, [36]
*M. plathytrix*	AJ698880, [36]	AJ698895, [36]
*M. matthey*	AJ698876, [36]	AJ698889, [36]
*M. musculoides*	AJ698875, [36]	AJ698890, [36]
*M. minutoides*	AJ875078, [84]	AJ875087, [84]
*M. indutus*	AJ698874, [36]	AJ698892, [36]
*M. haussa*	AJ698877, [36]	AJ698891, [36]
*M. caroli*^*a*^	AB033698, [35]	AJ698885, [36]
*M. cervicolor*^*a*^	AY057811, [82]	AJ698886, [36]
*M. cooki*^*a*^	AY057813, [83]	AJ698887, [36]
*M. fragilicauda*^*c*^	AB125779, [35]	AB125812, [35]
*M. famulus*^*c*^	AJ698872, [36]	AJ698884, [36]
*M. spicilegus*^*b*^	AF159397, [85]	AJ698882, [36]
*M. spretus*^*b*^	AB033700, [81]	AJ698883, [36]
*M. macedonicus*^*b*^	AB125770, [35]	AB125805, [35]
*M. cypriacus*^*b*^	FR751074, this study	__
*M. m. castaneus*^*b*^	AB125773, [35]	AB125806, [35]
*M. m. musculus*^*b*^	13838, [86]	AB125808, [35]
*M. m. domesticus*^*b*^	AB125774, [35]	__
*M. booduga*^*c*^	AB125761, [35]	AB125796, [35]
*M. terricolor*^*c*^	AB125776, [35]	AB125810, [35]

List of the species involved in the study with the accession number of the sequences and their references. The phylogenetic groups of the subgenus *Mus *are indicated.

a: Southeast Asian species

b: Paleartic species

c: Indian species

We used an ML approach to reconstruct the ancestral states of the rDNA clusters since we had no *a priori *knowledge on their mode of evolution. Using the previously determined reference tree (topology, branch length), the probability of each state (absent, pericentromeric or distal location) of the rDNA clusters was calculated at all nodes for each orthologous segment. This analysis was performed using R [37] and the function ACE (ancestral character estimation) from the package APE [38]. We used the ARD model (All-Rates-Different) where all change rates were different among the three rDNA cluster states.

### Association between rDNA clusters, breakpoints and centromeres

The number and positions of breakpoints were inferred from the chromosomal phylogeny derived by Veyrunes *et al. *[24], and comprised the sites involved in fissions, fusions, translocations and inversions. The events (breakpoints, loss or emergence of a centromere) were then mapped onto the reconstructed rDNA trees for each segment. The localization and co-occurrence of breakpoints and rDNA clusters were then determined.

The co-evolution between centromeres and rDNA clusters was next investigated by BayesTraits [39] using the previous phylogenetic framework. The correlated evolution between pairs of discrete binary traits (centromere, rDNA cluster) was analysed by ML. A matrix was constructed by coding 1/0 the presence/absence of a centromere and of an rDNA cluster for each orthologous segment and species. This analysis provided the likelihoods of an independent (4 parameters) and a dependent model (8 parameters) of evolution of centromeres and clusters. Likelihood ratio tests were performed to test the significance of the correlated evolution between centromeres and rDNA clusters with the statistics distributed as a chi square (df = 4).

## Results

### Overview

The chromosomal location of the 18S-28S rRNA genes for all species and subspecies is shown in Table 1. The distribution of rDNA clusters in this genus was highly variable ranging from 1 to 21 chromosome pairs. The Asian species harboured the highest number of clusters: 26-42 in M*us caroli, M. cervicolor, M. cooki, M. fragilicauda, M. famulus *and *M. pahari*. The exception was *M. platythrix *for which only three chromosome clusters were observed. This pattern contrasted markedly with the African subgenus *Nannomys *in which the number of clusters varied between two and 14.

The rDNA clusters were always located in the pericentromeric region (see insert Figure 1). This organization differed in two species (i) in *M. spretus*, the rDNA clusters were subtelomeric and (ii) in *M. terricolor*, the clusters were located between the telomere and the centromere [40]. The clusters were exclusively present on autosomal chromosomes with the exception of *M. caroli *which showed a signal on the X chromosome.

### Subgenus *Mus*

**Figure 2:**
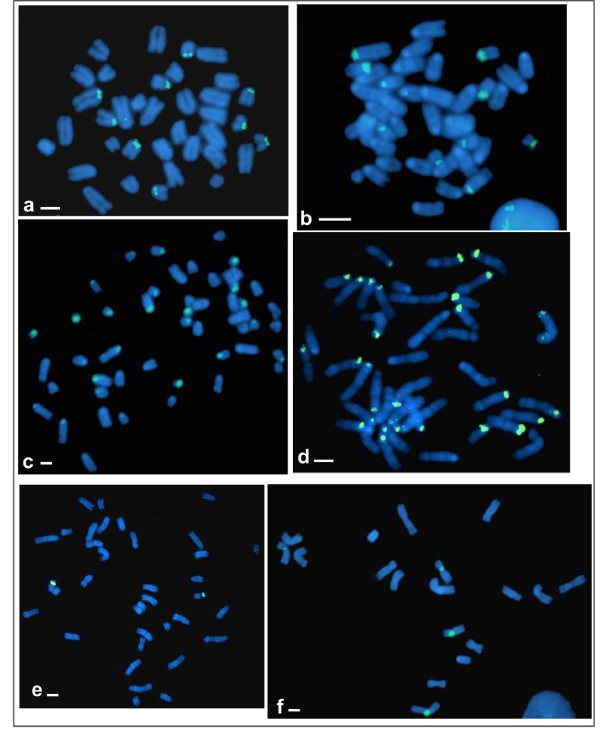
**FISH patterns using genomic clones of 18S-28S rDNA genes**. The chromosomal localization of rDNA clusters is shown in a) *Mus mattheyi*, b) *M. cypriacus*, c) *M. pahari*, d) *M. fragilicauda*, e) *M. haussa*, f) *M. musculoides*. Hybridization signals are visualized by FITC in green and metaphase spreads are counterstained with DAPI in blue. Scale bar indicates 10 μm.

Eight of the studied taxa showed a distribution of rDNA clusters that was identical to published data [see Table 1; [26,41,42]]. This included the rare subtelomeric position noted on chromosome 4 in *M. m. domesticus *[41]. New rDNA data are provided for six species (Figure 2). rRNA genes were present on all *M. caroli *chromosomes except the Y chromosome (i.e., all autosomes and the X). Among the Asian species, the rDNA clusters were located in the pericentromeric region of chromosomes 7-19 in *M. cooki*, of chromosomes 2-19 in *M. famulus*, and of chromosomes 4-19 in *M. cervicolor*. In *M. fragilicauda*, all autosomes harboured clusters. The 18S-28S rRNA genes were located on chromosomes 1, 4, 11, 15-19 in the endemic *M. cypriacus*. We detected variation in the number of clusters among the three specimens studied from different locations in Cyprus that showed five, seven and eight rDNA clusters respectively.

### Subgenus *Nannomys*

The 18S-28S rRNA genes were located in the pericentromeric regions of chromosomes: 1, 4, 8, 11, 13, 14 and 17 in *M. mattheyi *and chromosomes 14, 15, 17 in *M. indutus*. Only one signal was detected in *M. haussa *and this was on chromosome 15 which has a diagnostic pericentric inversion [22]. All autosomes in *M. musculoides *and the South African sample of *M. minutoides *result from centric fusions of ancestral acrocentric chromosomes [22]. The poor resolution of the centromeric areas in these metacentrics made the precise arm-localization of the hybridization signal problematic. Thus, the clusters were simply assigned to metacentrics: Rb(4.13) and Rb(8.15) in *M. musculoides*, and Rb(4.7), Rb(12.17), Rb(13.16) and Rb(14.15) in *M. minutoides*. The clusters were considered as present on both chromosomes of the metacentrics in the phylogenetic analyses.

### Subgenus *Coelomys*

*M. pahari *showed 34 signals on the centromeric regions of all but three chromosome pairs (6, 9 and X ).

### Subgenus *Pyromys*

Our results were similar to those of Matsubara *et al. *[26]: rDNA clusters were present on three chromosomes 5, 8 and 12.

### Inference of the ancestral state of clusters

**Figure 3:**
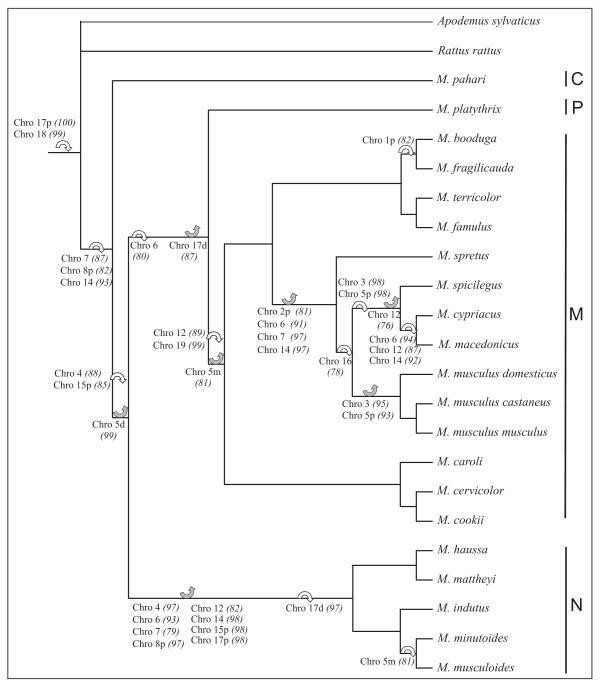
**Reference tree showing the changes in the state of clusters**. The appearance (white arrow) and disappearance (grey arrow) of clusters on the chromosomal segments are noted on the deepest node at which they occurred as well as the probability (%) of this event (in parentheses) (Chro = chromosome; p = proximal, d = distal, m = median). The subgenera are indicated on the right: C = *Coelomys*, P = *Pyromys*, M = *Mus*, N = *Nannomys*.

The results of the rDNA study allowed us to identify a total of 30 clusters in the genus, 27 of which were located in the pericentromeric area and three were telomeric. These clusters were mapped onto the 30 orthologous segments of the ancestral *Mus *karyotype [24] (Figure 1). The phylogenetic distribution of the rDNA clusters was established for each of the orthologous segments except one, since an rDNA cluster was never observed on the segment corresponding to chromosome 10d (see additional files 1, 2, 3 and 4). At each node of the tree, the ACE analysis assigned a probability (0-100%) for the absence/presence of a cluster at a pericentromeric/subtelomeric position. From the probability distribution of all segments and nodes, we determined a threshold probability value of 75%, i.e., each state associated with a probability ≥75% was considered as known. Using this threshold value, the state of the rDNA clusters for a segment could be ascertained for 65% of the nodes over all segments i.e., a cluster was absent (or present) in 42% (or 23%) of the nodes. Among the 29 segments, the state of the cluster could not be determined for four of them since all nodal character states had a probability of 50%. In seven additional segments, clusters were never present, whereas for one segment (Chro 18, see Figure 3), a cluster was present at all nodes. Overall, the results indicated that the rDNA clusters showed a high lability: three insertions and 10 deletions could be validated in the deeper nodes and 11 insertions/25 deletions in the terminal branches. The deletions occurred in all subgenera, whereas 86% of the insertions concerned the subgenera *Coelomys *and *Mus*. The recurrence of events on segments 6, 7, 12 and 14 (41% of insertions and deletions) is noteworthy. For example, the cluster on the orthologous segment to chromosome 14 was present at the ancestral node of the genus *Mus*. This cluster disappeared at the nodes leading to the subgenus *Nannomys *and to the European species of the subgenus *Mus *(*M. spretus, M. musculus, M. spicilegus, M. cypriacus, M. macedonicus*), and then reappeared at the node leading to *M. cypriacus + M. macedonicus *(Figure 3).

### Association between breakpoints, rDNA clusters and centromeres

**Figure 4:**
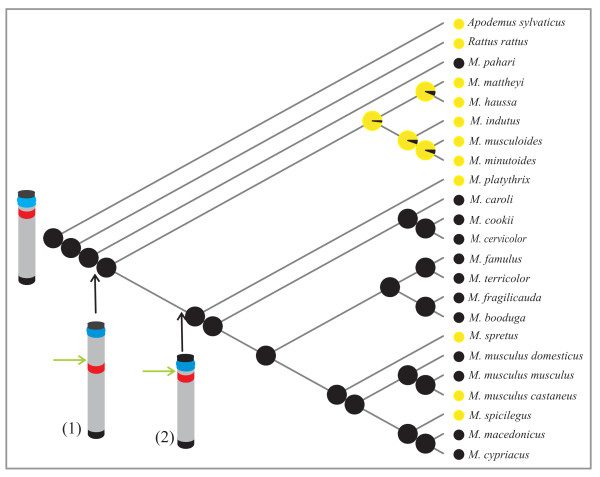
**Reference tree indicating the state probability of the cluster on the orthologous segment 17p**. At each node, the probability of the state of the rDNA cluster is reported as a pie: black = presence, yellow = absence. The ancestral chromosome 17p is drawn at the base of the tree and the position of two rearrangements is indicated: (1) a translocation occurred, leading to the addition of a new centromere and the loss of the previous one, the rDNA cluster becomes interstitial. (2) Following a fission event, a new centromere is subsequently reacquired in the original position; the rDNA cluster is again located in a pericentromeric region. The breakpoints are indicated by green arrows, centromeres are in blue and rDNA clusters in red.

The 42 breakpoints identified by Veyrunes *et al. *[24] were mapped onto the ancestral karyotype as well as the location of the rDNA clusters and centromeres observed in the genus (Figure 1). The comparative distribution of these three genomic structures highlighted that 26/30 of the rDNA clusters occurred in the vicinity of breakpoint regions and that 96% of these clusters were also associated with a centromere. Thus, more than half (25/42) of the rearrangement breakpoints in the genus are localized at sites where both an rDNA cluster and a centromere were present. The phylogenetic analyses performed herein together with the published chromosomal phylogeny [24] provided the opportunity to investigate the temporal dynamics of these genomic structures. In other words, was an rDNA cluster present on the orthologous segment when the rearrangement occurred? When the breakpoints and centromeres were mapped onto the rDNA trees of each orthologous segment, the results showed that the state of clusters for nine breakpoints could not be determined, and that 13 breakpoints did not involve a cluster or a centromere. In all, eight breakpoints occurred in a region where an rDNA cluster as well as a centromere were present. In two cases, the rearrangement resulted in the loss of both the centromere and the cluster suggesting that the break occurred at the distal end of the cluster (see Figure 4). In the six remaining cases, the break was situated between the centromere and the rDNA cluster. These rearrangements led to the loss of the centromere, four of which were subsequently reacquired. Discounting the undetermined clusters, these data suggest that 24% of breakpoints occurred on either side of a cluster.

Given the predominantly pericentromeric location of rDNA clusters and the co-occurrence of centromeres and clusters at breakpoints, the evolutionary association between centromeres and rDNA clusters was tested. The results of this analysis are summarized in Table 3. Of the 30 orthologous segments, one showed no rDNA cluster and one exhibited a cluster at a telomeric location only. These segments were thus eliminated from the analysis. Of the 28 remaining segments, four showed significant evidence of co-evolution between centromeres and rDNA (p-value = 0.03-0.04). For example, the centromere of the segment corresponding to chromosome 19 appeared at the ancestral node of the subgenus *Mus*. At the same node, an rDNA cluster appeared on this segment and persisted in all species of the subgenus (see Figure 3). In five other cases, co-evolution approached significant values (p = 0.06-0.11). Finally, in the remaining 19 segments, the independent evolution of centromeres and clusters could not be rejected. This is illustrated by the evolution of the rDNA cluster on the segment orthologous to chromosome 7 (see Figure 3). A centromere is always present on this segment but the cluster which was present on the ancestral node to the genus, is lost on the node leading to *Nannomys *and on the node leading to the European species (M*us spretus, M. musculus, M. spicilegus, M. cypriacus, M. macedonicus*).

**Table 3:** Test of the evolutionary association between centromeres and cluster

Chromosomal segment	lLn (Independent)	lLn (Dependent)	ΔlnL	p-value
19	-12.9	-7.4	10.9	0.03
8p	-21.7	-16.3	10.8	0.03
17p	-19.1	-14	10.1	0.04
4	-18.7	-13.5	10.3	0.04
15p	-17.5	-13	8.9	0.06
17d	-14.3	-10	8.5	0.07
2d	-9.7	-5.4	8.5	0.07
6	-19.5	-15.3	8.3	0.08
13p	-21.3	-17.5	7.6	0.11
15d	-7.9	-5.4	5.1	0.28
8d	-7.9	-5.4	5.1	0.28
5p	-16.3	-13.8	5	0.29
5d	-10.7	-8.5	4.4	0.35
10p	-19.4	-17.7	3.4	0.49
5m	-6.9	-5.47	3	0.56
16	-15.1	-13.77	2.8	0.6
13d	-8.9	-7.5	2.8	0.59
1d	-8.9	-7.5	2.8	0.59
11d	-10.6	-9.25	2.7	0.61
18	-16.7	-15.45	2.5	0.64
9	-17.9	-17	1.9	0.76
12	-18.3	-17.4	1.7	0.78
1p	-15.5	-15.1	0.9	0.93
3	-15.9	-15.5	0.8	0.94
2p	-15	-14.6	0.7	0.95
11p	-14.9	-14.5	0.7	0.95
14	-13.9	-13.9	0.1	1
7	-11.1	-10.9	0.3	0.99

For each orthologous segment (p = proximal, d = distal, m = median), the log-likelihood of the models with dependent or independent evolution between centromeres and clusters is provided, along with the difference in log-likelihood between them, and the resulting likelihood ratio test p-value.

## Discussion

### Extensive variation in number and location of rDNA clusters

This study is the first to report such an impressive variation in number and localization of rDNA clusters within a mammalian genus. The rDNA clusters in the *Mus *species were present on 1 to 21 pairs of chromosomes with important numerical differences between the taxa of the different subgenera. Overall, the subgenera *Coelomys *and *Mus *possessed a much larger number of clusters than species within the subgenera *Nannomys *and *Pyromys*. This difference is reflected by that of the diploid numbers since the karyotypes in the former subgenera have more chromosomes (2n = 48, 40) than those in the latter (2n = 18 to 36). As there is generally only one cluster per chromosome pair, a lower diploid number entails fewer chromosomes and thus a reduction in the number of available sites for rRNA genes. Notwithstanding the relation between rDNA cluster and chromosome number, however, variation in the number of clusters was also considerable within the subgenus *Mus*, although all species share the same 2n = 40 karyotype. In this group, the Palaearctic species have less than half the number of clusters present in the Asian and Indian species. Given that the origin of *Mus *lies in southern Asia [35], the decrease in cluster number may be compatible with the loss of rDNA sites by stochastic processes during the westward colonization of the ancestral taxa.

In the genus *Mus*, the high number of clusters observed raises questions on the processes involved in maintaining such a large number of repeats. Whereas cellular life requires a minimum of one cluster of rRNA genes to construct the ribosome, our results suggest that there may be no upper constraint on the maximal number of clusters in a genome. In a study on rDNA cluster number variation in 40 species of rodents, a mean of 4.2 chromosome pairs carried rDNA clusters (range 1 to 5 [16]). This is far below the value observed in the present study (mean: 10.1; range 1 to 21). However, the number of clusters provides no information on the number of copies within a cluster, or on their transcriptional activity. It is possible that when many clusters are present, the number of repeat units at each chromosomal site may in fact be small (not studied). With respect to transcriptional activity of repeats within and between clusters, cytogenetic methods involving Ag-staining (silver nitrate stain) may be useful. This approach identifies rRNA genes that were transcribed during the previous interphase. Comparison between published Ag-staining data *vs *our own FISH analyses for nine *Mus *species revealed discrepancies in some taxa [41-43]. This was the case for example in *M. caroli *(15 Ag- staining/20 FISH), *M. cervicolor *(9/19) and *M. fragilicauda *(16/19). Differences such as these have similarly been observed in other vertebrate groups such as teleosts, bats, and horses [44-47]. Determining the frequency of these silent rRNA genes on a larger sample of mice will provide clues to their regulation patterns [48].

Another notable characteristic of the rDNA clusters in the genus *Mus *is their almost exclusive pericentromeric location since they were subtelomeric in only one species (*M. spretus*). In rodents, both positions are commonly observed (58% pericentromeric/31% distal *vs *11% interstitial) [25,27,49-56]. Few mammal species (8/126) exhibit clusters in an interstitial position and in several cases, these result from chromosomal rearrangements [50,57]. It should be noted that none of the extant *Mus *species harboured rDNA clusters in an interstitial position, although the phylogenetic reconstruction inferred four instances in which they occupied a transient interstitial position following a rearrangement (Figure 4). The low frequency of these clusters suggests that an interstitial location may not be evolutionary stable and this may be due to two possibilities: (i) the location may be deleterious, or, (ii) as suggested by our results, it may contribute to genomic instability and predispose the chromatin to centromere formation [58].

The location of clusters between *Mus *species differed, each taxon showing its own chromosomal distribution. Thus, the chromosomal distribution of rDNA clusters appears as a useful cytogenetic marker (number and position on the chromosome) to discriminate species [59]. However, as our results have shown that rDNA clusters may be labile, their use in inferring orthologous chromosomal sites between species must be treated with caution [e.g. Anura; [60]].

### Do rDNA clusters contribute to rearrangements?

Our study is the first to reconstruct the phylogeny of rDNA clusters. By combining this original approach and a published chromosomal phylogeny, the evolutionary relationship between rDNA clusters and rearrangements could be evaluated and the association with the accelerated rate of genome repatterning of the genus *Mus *explored. Previous studies have underscored the pivotal role of centromere change in the high rate of chromosomal evolution in this group of rodents [13,24]. The phylogenetic analyses performed in this present study provided a unique opportunity to reconstruct the timing and infer the position of breakpoints relative to that of the centromere and the rDNA site. Two patterns were evidenced. First, we were able to determine that 24% of the breakpoints occurred at the proximal or distal end of the rDNA clusters. The corresponding rearrangements all involved the coincidental loss of linked centromeres, some of which subsequently re-emerged in the same position. This association is further supported by the significant co-evolutionary behaviour observed between some of the rDNA clusters and centromeres suggesting that the joint presence of these two genomic structures may lead to genome instability and predispose to chromosomal rearrangements. Second, we identified an impressive rate of rDNA cluster change (deletions and insertions), most of which somewhat surprisingly occurred in the subgenus *Mus*, an evolutionary clade in which all species share the same chromosomal complement. These results suggest that rDNA clusters can move from one chromosome pair to another with no other modifications of the karyotype.

A role of rDNA genes as promoters of genome reorganization, particularly when located in the pericentromeric region, has previously been the focus of studies on the mechanisms of centric or Robertsonian (Rb) fusions [61,62]. This type of rearrangement involves the joining by the centromere of two non-homologous chromosomes. The rationale of a direct or indirect role in the Rb fusion was based on the fact that rDNA clusters group together during interphase to form one or several nucleoli. The physical proximity between rDNA-bearing chromosomes in interphase was predicted to increase the probability of their being involved in Rb fusions. Strikingly, this rearrangement is prominent within two of the present subgenera studied herein. In two of the samples studied of the subgenus *Nannomys*, all chromosomes were the product of Rb fusions [22]. The hybridization signal of the rDNA probe seemed to co-localize with the junction between the two chromosomal arms, suggesting that at least one of the breakpoints may have occurred in or close to the rDNA cluster. In the subgenus *Mus*, populations within two taxa carry Rb fusions: *M. m. domesticus *and *M. terricolor*. In the latter, only two rare Rb fusions are documented [40]. In contrast, *M. m. domesticus *shows a very high diversity of populations carrying different numbers and combinations of fusions, although only five pairs of chromosomes harbour pericentromeric rDNA clusters [[63], this study]. The fusion mechanism is well documented in *M. m. domesticus *and has been shown to involve breaks in the centromeric satellite sequences [64] and not in the rDNA clusters which are conserved intact. In addition, the prediction of a higher involvement of the rDNA-bearing chromosomes in the Rb fusions was not confirmed in the house mouse, as a statistical analysis indicated that they had the lowest frequency of fusion [65]. These findings suggest that centric fusions may involve different sequences in different genomes, and determining the precise mechanism involved requires high resolution sequence analysis.

### Is there a functional association between centromeres and rDNA clusters?

Our results highlight two characteristic features of rDNA cluster distribution in the genus *Mus*: (i) rDNA clusters are preferentially located in pericentromeric regions, and (ii) a change in chromosomal distribution of rDNA clusters always occurs between pericentromeric regions of different chromosomes. This physical linkage between rDNA clusters and centromeres may be driven by their genomic structures, since both are subject to sequence homogenization. In the case of rRNA genes, several studies have underscored the high degree in sequence similarity within and between genomes [15]. Two models of evolution have been proposed to account for this observation. These are the Birth and Death model and the concerted evolution model [66,67]. By comparing the level of intragenomic variation of rDNA sequences, Ganley and Kobayashi [68] were able to confirm that rRNA genes evolved via concerted evolution. This model involves two mechanisms of sequence homogenization: gene conversion and non-homologous recombination. Gene conversion corresponds to a non-reciprocal transfer of an allelic difference from one chromosome to its homologue, whereas non-homologous recombination consists in a recombination event between non-sister chromatids. In humans, studies have shown that rDNA clusters were involved in meiotic rearrangements at a frequency >10% per cluster and per meiosis [69], and that the regulatory and coding sequences were highly homogenized [70]. Similar observations of high sequence homogeneity exist for both subtelomeric and particularly centromeric regions in several mammalian species [71-73].

**Figure 5:**
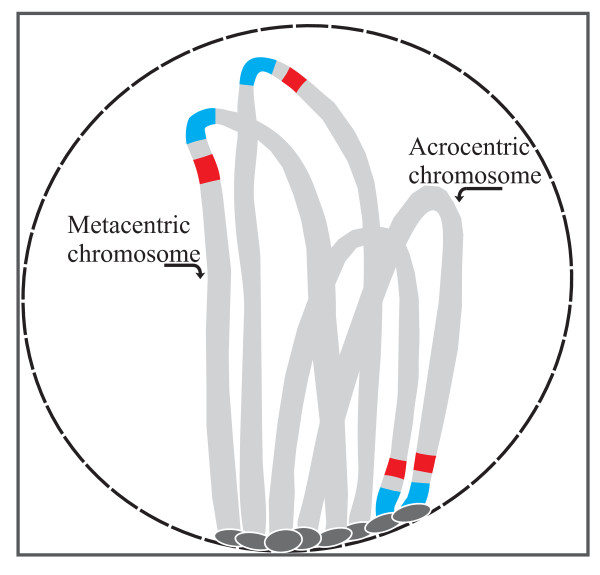
**The bouquet organization of chromosomes during the prophase [adapted from 74]**. All telomeres are attached to the nuclear envelope. The physical proximity between telomeres (grey), centromeres (blue) and rDNA clusters (red) of acrocentric chromosomes may facilitate their sequence homogenization by non-homologous recombination.

How and when would concerted evolution take place? During early prophase, all the chromosomes migrate into one area of the nucleus and adopt a particular orientation known as the bouquet in which all telomeres attach to the nuclear membrane (Figure 5) [74]. Double-strand breaks also appear at this stage that are programmed to be repaired by recombination be it by reciprocal exchange (cross-over) or gene conversion [75]. Thus, the formation of the bouquet provides the physical opportunity for concerted evolution to occur between similar sequences on non-homologous chromosomes (such as centromeres, and the associated rDNA genes [71,72,76]). Reciprocally, the presence of clusters could increase the rate of non-homologous recombination in the pericentromeric or subtelomeric regions. Several consequences of rDNA evolution are expected. First, rDNA clusters would be predicted to localize preferentially in centromeric or subtelomeric regions. This is in agreement with available data in mammalian species. Second, whereas the telomere orientation in the bouquet obviously leads to the close proximity between centromeres on acrocentric chromosomes during meiosis, this is less evident where metacentric chromosomes are involved (Figure 5). This difference is likely to influence the rate of homogenization between centromeres (and linked rRNA genes) of acrocentric *vs *metacentric chromosomes. Such a pattern has in fact been confirmed in humans and recently in the pig in which the percentage similarity of centromeric sequences is higher in acrocentric than metacentric chromosomes [71,77]. Finally, the mechanism of concerted evolution in a bouquet context paves the way for the occurrence of exchanges of rDNA clusters between non-homologous chromosomes.

## Conclusion

In conclusion, our results agree with a main outcome of recent studies indicating that breakpoints overlap with a diversity of repetitive families among different groups [5], which thus may include rDNA clusters. The present analysis on the dynamics of rDNA clusters within the genus *Mus*, has revealed an impressive variation in the number and location of clusters between taxa, and highlighted the strong evolutionary relationship between rDNA clusters and centromeres. Both of these genomic structures coincide with breakpoints in the genus *Mus*, suggesting that the accumulation in the centromeric region of a large number of repeats subject to concerted evolution may contribute to the high rates of chromosome repatterning observed in this group. However, the high rate of rDNA change observed in the chromosomally invariant subgenus *Mus *indicates that the presence of these sequences is insufficient to lead to genome instability. Thus, an additional factor would be required to trigger evolutionary plasticity. Emerging clues now point to modifications of the epigenetic state of DNA, particularly of interspersed repeats, as a prime source of instability [1,6,78]. In plants and insects, it has been shown that transposable elements may be associated with rDNA clusters, and thus, may be responsible for changes in their chromosomal distribution [15,79]. However, no direct association between rRNA genes and transposable elements has so far been described in mammals. Although this study deals with large-scale rearrangements and low-resolution breakpoints, our results may serve to focus genomic investigations on the factors triggering genome plasticity and evolution.

## Abbreviations

SINE: short interspersed nuclear element; LINE: long interspersed nuclear element; LTR: long terminal repeat; FITC: fluorescein isothiocyanate; FISH: fluorescence *in situ *hybridization; ML: maximum likelihood

## Authors' contributions

BC carried out the laboratory work, and participated in all analyses. JC participated in the acquisition of data, and drew the figures. FV participated in the design of the study. EJPD participated in the analyses. JBD conceived the study. BC and JBD wrote the paper. All authors read and approved the final manuscript.

## Supplementary Material

Additional file 1 **ML trees with the consensus topology for the orthologous segments 1p to 5d**. The probability of the state of the rDNA cluster is shown as a pie at each node. The absence of a cluster is indicated in yellow, the presence in a pericentromeric region in black and the presence in a distal region in red. Click here for file

Additional file 2 **ML trees with the consensus topology for the orthologous segments 6 to 12d**. The probability of the state of the rDNA cluster is shown as a pie at each node. The absence of a cluster is indicated in yellow, the presence in a pericentromeric region in black and the presence in a distal region in red. Click here for file

Additional file 3 **ML trees with the consensus topology for the orthologous segments 13 to 17d**. The probability of the state of the rDNA cluster is shown as a pie at each node. The absence of a cluster is indicated in yellow and the presence in a pericentromeric region in black. Click here for file

Additional file 4 **ML trees with the consensus topology for each orthologous segment from 18 to 19**. The probability of the state of the rDNA cluster is shown as a pie at each node. The absence of a cluster is indicated in yellow, the presence in a pericentromeric region in black and the presence in a distal region in red. Click here for file
