# Supplementary figures and images for: Are ribosomal DNA clusters rearrangement hotspots? A case study in the genus Mus (Rodentia, Muridae)
Source: BMC Evol Biol. 2011 May 13;11:124. doi: 10.1186/1471-2148-11-124 (PMC3112088; doi:10.1186/1471-2148-11-124)

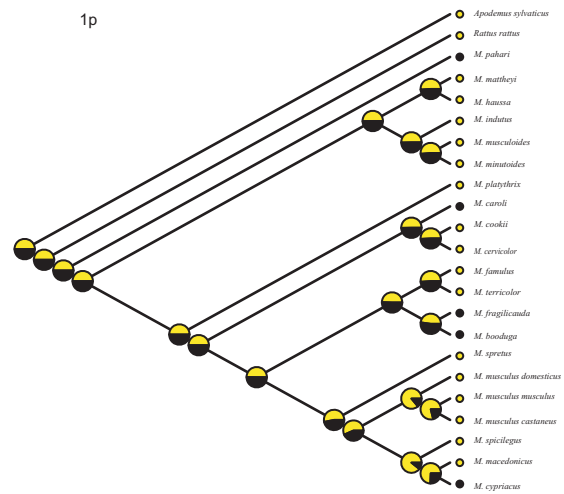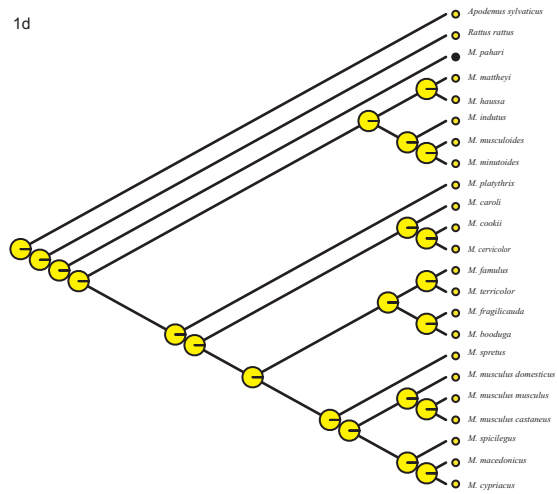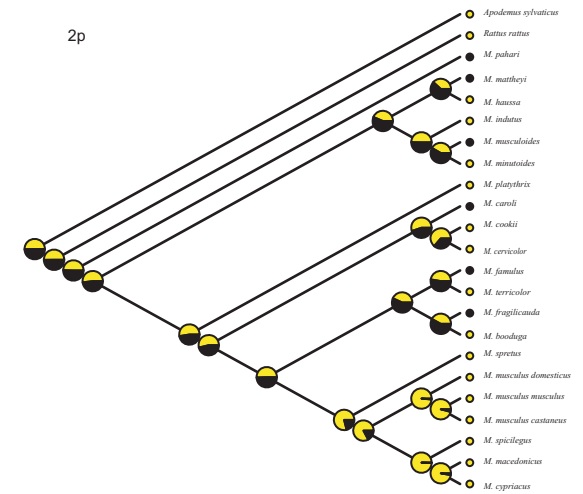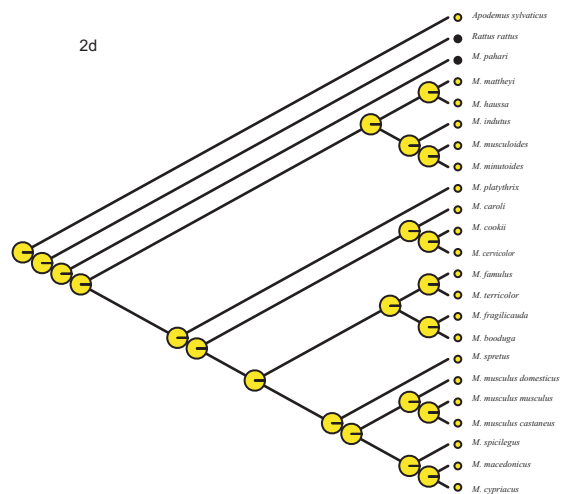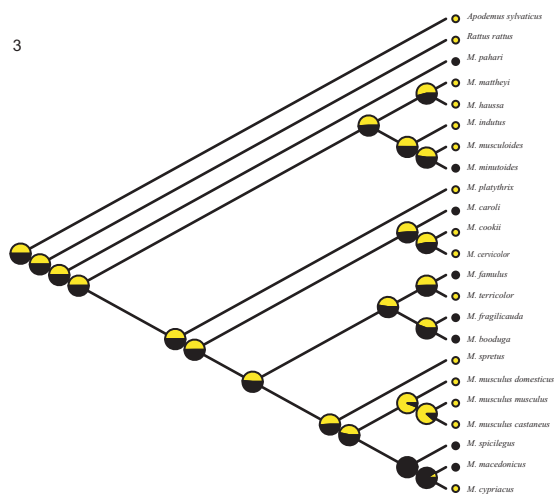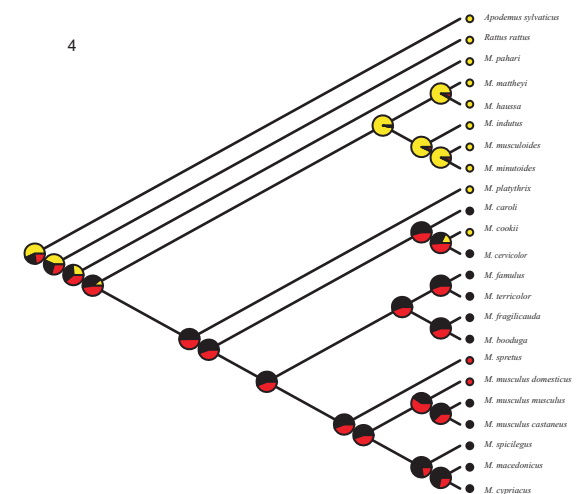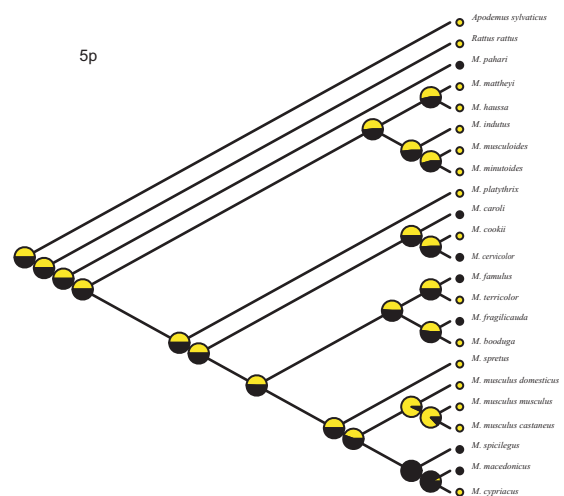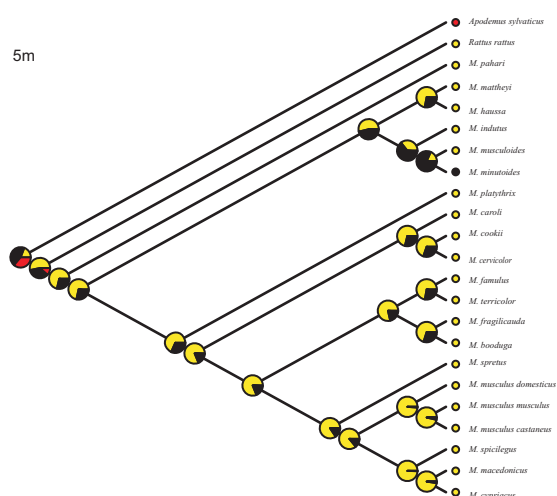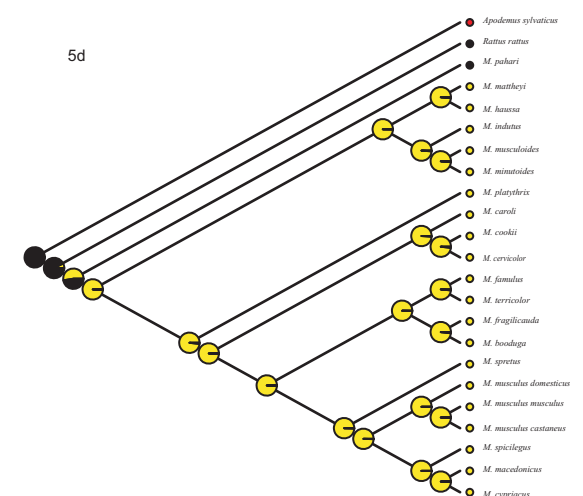

Supplement: Additional file 1 — ML trees with the consensus topology for the orthologous segments 1p to 5d. The probability of the state of the rDNA cluster is shown as a pie at each node. The absence of a cluster is indicated in yellow, the presence in a pericentromeric region in black and the presence in a distal region in red. [file 1471-2148-11-124-S1.PDF]

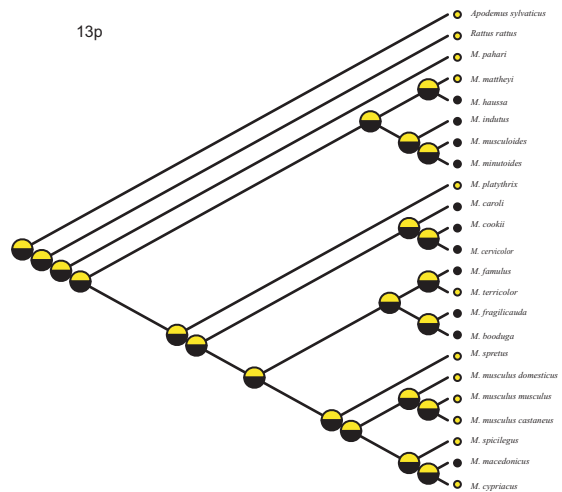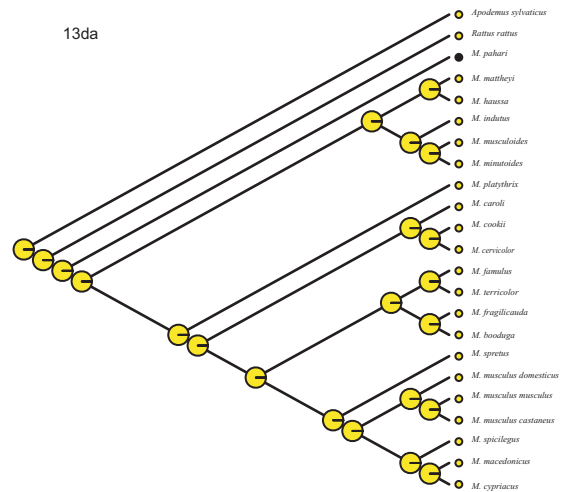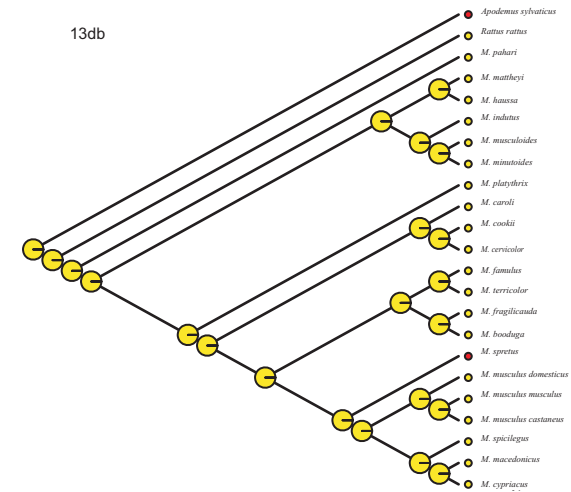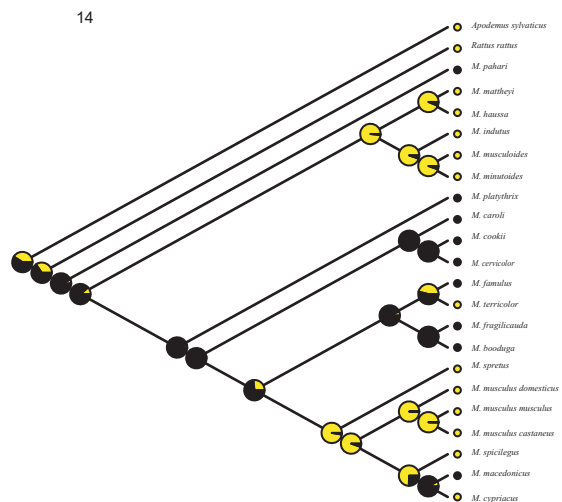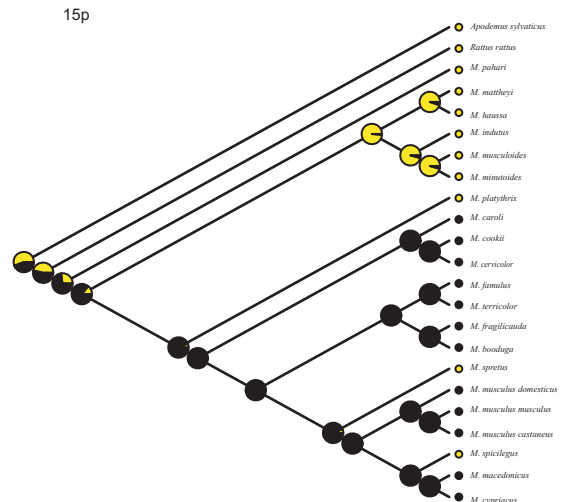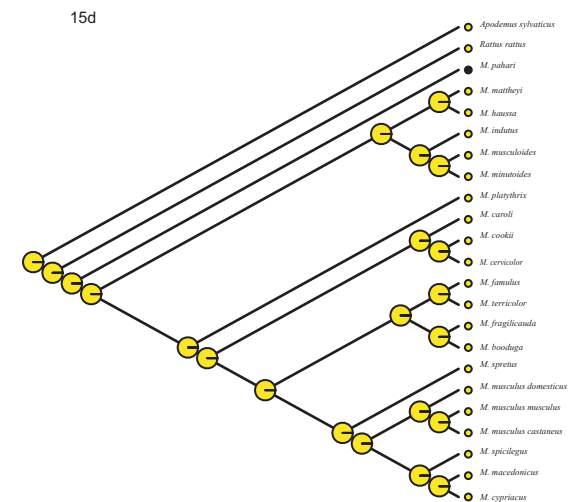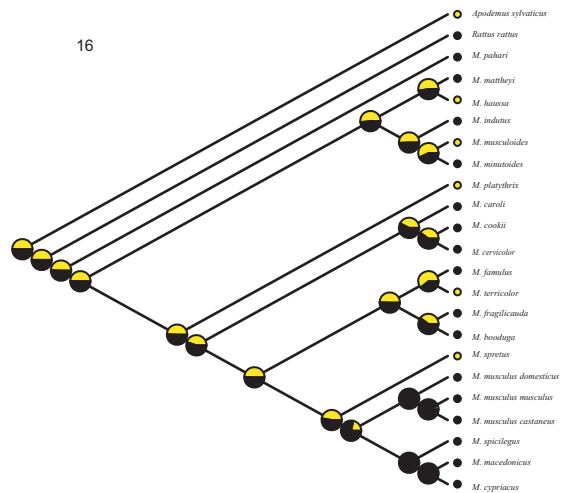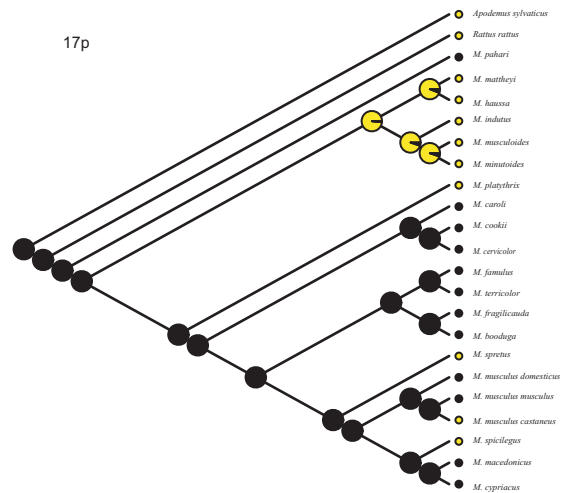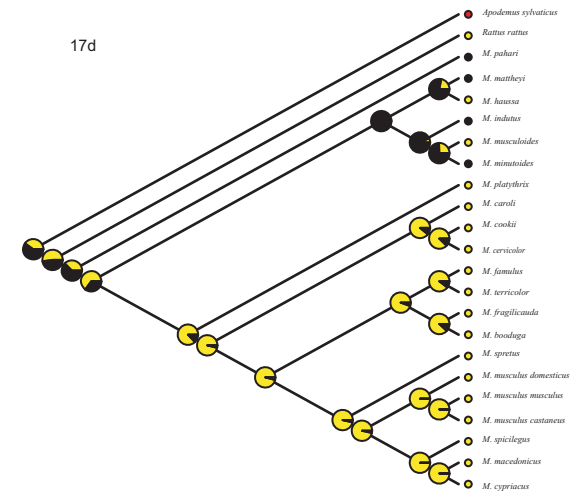

Supplement: Additional file 3 — ML trees with the consensus topology for the orthologous segments 13 to 17d. The probability of the state of the rDNA cluster is shown as a pie at each node. The absence of a cluster is indicated in yellow and the presence in a pericentromeric region in black. [file 1471-2148-11-124-S3.PDF]

18

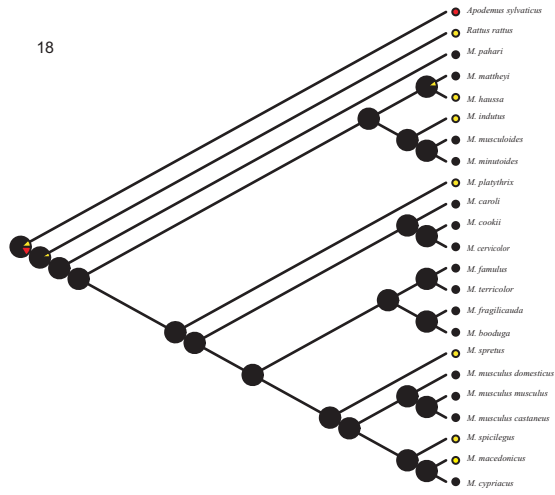

19

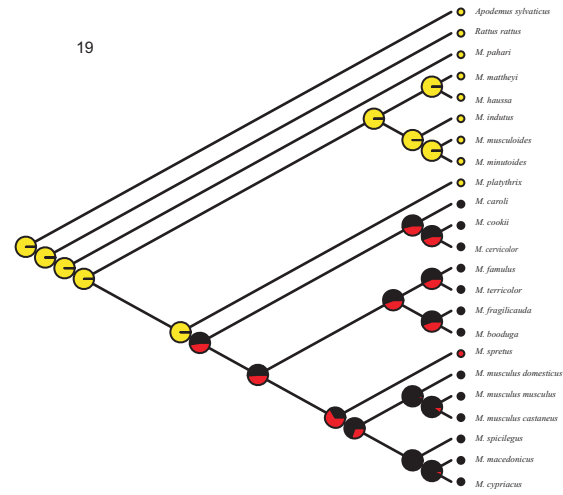

Supplement: Additional file 4 — ML trees with the consensus topology for each orthologous segment from 18 to 19. The probability of the state of the rDNA cluster is shown as a pie at each node. The absence of a cluster is indicated in yellow, the presence in a pericentromeric region in black and the presence in a distal region in red. [file 1471-2148-11-124-S4.PDF]
