# Supplementary material for: Are ribosomal DNA clusters rearrangement hotspots? A case study in the genus Mus (Rodentia, Muridae)
Source: BMC Evol Biol. 2011 May 13;11:124. doi: 10.1186/1471-2148-11-124 (PMC3112088; doi:10.1186/1471-2148-11-124)
Supplement: Additional file 2 — ML trees with the consensus topology for the orthologous segments 6 to 12d. The probability of the state of the rDNA cluster is shown as a pie at each node. The absence of a cluster is indicated in yellow, the presence in a pericentromeric region in black and the presence in a distal region in red. [file 1471-2148-11-124-S2.PDF]

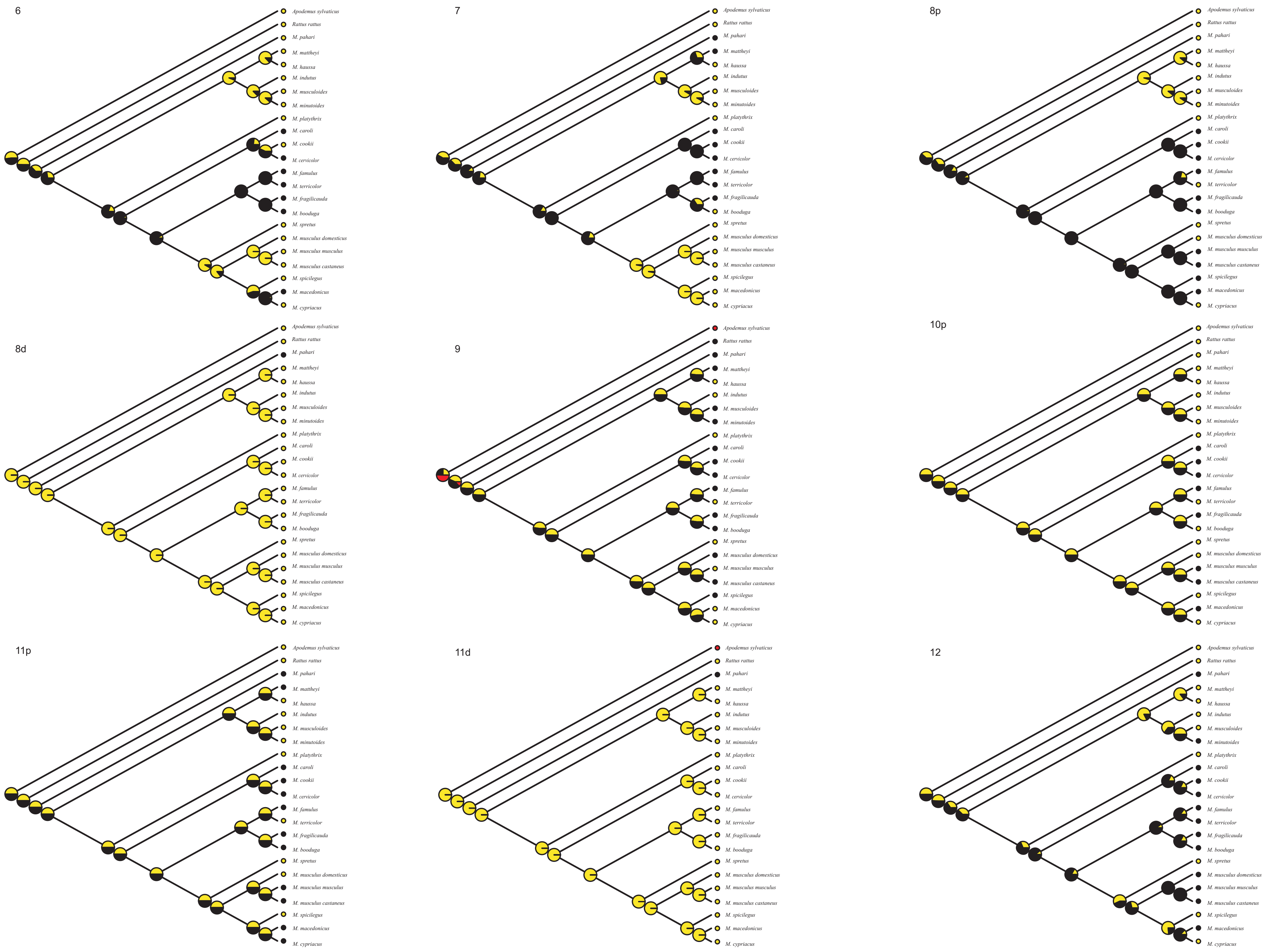
6
7
8p
8d
9
10p
11p
11d
12
Apodemus sylvaticus
Rattus rattus
M. pahari
M. mattheyi
M. haussa
M. indutus
M. musculoides
M. minutoides
M. platythrix
M. caroli
M. cookii
M. cervicolor
M. famulus
M. terricolor
M. fragilicauda
M. booduga
M. spretus
M. musculus domesticus
M. musculus musculus
M. musculus castaneus
M. spicilegus
M. macedonicus
M. cypriacus
